# Comparison of regional cerebral oxygen saturation during one-lung ventilation under desflurane or propofol anesthesia: A randomized trial

**DOI:** 10.1097/MD.0000000000030030

**Published:** 2022-10-14

**Authors:** Keishu Hayashi, Yuko Yamada, Takuma Ishihara, Kumiko Tanabe, Hiroki Iida

**Affiliations:** a Department of Anesthesiology and Pain Medicine, Gifu University Graduate School of Medicine, Gifu, Japan; b Innovative and Clinical Research Promotion Center, Gifu University Hospital, Gifu, Japan; c Anesthesiology and Pain Relief Center, Central Japan Medical Center, Minokamo, Japan.

**Keywords:** cerebral blood flow, cerebral oxygen saturation, near-infrared spectroscopy, one-lung ventilation, oxidative stress, thoracic surgery

## Abstract

During one-lung ventilation (OLV), deterioration of pulmonary oxygenation reduces arterial oxygen saturation and cerebral oxygen saturation (rSO_2_). However, oxidative stress during OLV causes lung injury, so the fraction of inspiratory oxygen (FiO_2_) should be kept as low as possible. We investigated the changes in rSO_2_ under propofol or desflurane anesthesia while percutaneous oxygen saturation (SpO_2_) was kept as low as possible during OLV.

**Methods:**

Thirty-six patients scheduled for thoracic surgery under OLV in the lateral decubitus position were randomly assigned to propofol (n = 19) or desflurane (n = 17) anesthesia. FiO_2_ was set to 0.4 at the start of surgery under two-lung ventilation (measurement point: T3) and then adjusted to maintain an SpO_2_ of 92% to 94% after the initiation of OLV. The primary outcome was the difference in the absolute value of the decrease in rSO_2_ from T3 to 30 minutes after the initiation of OLV (T5), which was analyzed by an analysis of covariance adjusted for the rSO_2_ value at T3.

**Results:**

The mean rSO_2_ values were 61.5% ± 5.1% at T3 and 57.1% ± 5.3% at T5 in the propofol group and 62.2% ± 6.0% at T3 and 58.6% ± 5.3% at T5 in the desflurane group. The difference in the absolute value of decrease between groups (propofol group − desflurane group) was 0.95 (95% confidence interval, [−0.32, 2.2]; *P* = .152).

**Conclusions:**

Both propofol and desflurane anesthesia maintain comparable cerebral oxygenation and can be used safely, even when the SpO_2_ is kept as low as possible during OLV.

## 1. Introduction

One-lung ventilation (OLV) is an indispensable anesthetic procedure for thoracic surgery. During OLV, the collapsed and unventilated lung still has perfusion that can cause intrapulmonary shunting, leading to impaired oxygenation and hypoxemia. Although a high fraction of inspiratory oxygen (FiO_2_) tends to be set to avoid hypoxemia during OLV, high FiO_2_ levels also increase the risk of postoperative respiratory complications and 30-day mortality.^[1,2]^ Furthermore, oxidative stress during OLV causes lung injury, which is exacerbated by a higher FiO_2_.^[3]^ The FiO_2_ should thus be kept as low as possible while maintaining the percutaneous oxygen saturation (SpO_2_) level within the appropriate range.^[4]^

There is some concern about an insufficient oxygen supply to vital organs, especially the central nervous system, during OLV. Cerebral oxygenation is evaluated by the regional cerebral oxygen saturation (rSO_2_) measurement. It has been reported that the rSO_2_ declines during OLV despite the SpO_2_ being above 90%, which may induce postoperative cognitive dysfunction.^[5–9]^ Sungur et al^[8]^ reported that preventing cerebral desaturation might reduce postoperative cognitive dysfunction.

Recently, for adult patients, desflurane has been more commonly used as an anesthetic agent than sevoflurane because of its lower in vivo metabolic rate and faster emergence from general anesthesia. Desflurane has similar oxygenation, shunt fraction, and hemodynamics to sevoflurane during OLV.^[10]^ Although arterial oxygenation during OLV has been reported to be reduced with desflurane-remifentanil anesthesia in comparison to propofol-remifentanil anesthesia, desflurane is nevertheless still frequently used as an anesthetic agent.^[11]^

In general, both propofol and desflurane reduce the cerebral metabolic rate for oxygen (CMRO_2_), although their effects on the cerebral blood flow (CBF) are not the same. Propofol has a cerebrovascular contractile effect and reduces both the CMRO_2_ and CBF.^[12]^ Of note, the reduction in CBF outweighs the reduction in CMRO_2_ due to propofol, resulting in an overall reduction in the CBF/CMRO_2_ ratio.^[13–15]^ Conversely, volatile anesthetic agents, such as desflurane, have a dose-dependent cerebrovascular dilator effect, which causes an increased CBF at concentrations >1 minimal alveolar concentration (MAC).^[16–18]^ As such, the cerebral oxygen balance during OLV can be impaired under propofol anesthesia in comparison to volatile anesthetics. A previous study further showed that cerebral oxygen desaturation evaluated based on the jugular bulb venous oxygen saturation (SjO_2_) occurred more frequently under propofol anesthesia than under sevoflurane anesthesia during OLV, with an FiO_2_ of 0.5 and SpO_2_ of approximately 95%.^[19]^ To our knowledge, no previous study has compared cerebral oxygenation during OLV under desflurane and propofol anesthesia.

We hypothesize that cerebral oxygenation is more markedly impaired during OLV under propofol anesthesia than under desflurane anesthesia. The present study therefore compared the effects of propofol and desflurane on cerebral oxygenation measured by rSO_2_ during OLV to avoid a high FiO_2_ level in patients undergoing thoracic surgery.

## 2. Materials and Methods

### 2.1. Study design and participants

This single-center, single-blinded, randomized clinical trial with parallel groups took place in Gifu University Hospital. The study protocol was approved by the Institutional Review Board of The Gifu University Hospital (decision number: 2019-274, approval date: March 16, 2020) and was registered at University hospital Medical Information Network Center (identifier: UMIN000039403, resister date: March 4, 2020).

We evaluated patients of ≥20 years of age who were scheduled for elective thoracic surgery requiring OLV in the lateral decubitus position at Gifu University Hospital from March to October 2020. Cases involving esophagectomy, robot-assisted thoracic surgery, lung resection for pneumothorax, surgery with carbon dioxide gas insufflation for pneumothorax and surgery with an expected duration of OLV < 30 minutes were not included in the analysis.

Participants who did not meet the exclusion criteria were asked to give their written informed consent before enrollment. The exclusion criteria were a medical history of lobectomy or segmentectomy, cerebrovascular disease, coronary artery disease, heart failure, or interstitial pneumonia (including suspicious cases); an American Society of Anesthesiologists physical status classification of ≥Ⅲ for any other reason; contraindication of study drugs; and any other reason deemed to render the participant inappropriate for intervention.

This study was conducted in accordance with the Consolidated Standards of Reporting Trials (CONSORT) 2010 statement.

### 2.2. Randomization and masking

The data manager used the R software program, version 3.6.3 (www.r-project.org), to create a randomization sequence with 1:1 allocation, a mixed block size of 2 or 4, and no stratification. Researchers were not informed of the block size throughout the trial. The researchers assigned patients to either the propofol or desflurane group using sealed envelopes prepared according to a pregenerated allocation table. Sequence generation and randomization envelope preparation were performed by the data manager, independent of the researchers who had no further role in the trial. The patients were not informed of the treatment allocation.

### 2.3. Procedures

The 36 total patients were randomly allocated to the desflurane or propofol group. After arriving in the operating room, a cerebral oximeter probe and electroencephalograph sensor were placed on the forehead of the subject and connected to a regional cerebral oximetry system (O3 regional oximetry; Masimo, Irvine, CA) and electroencephalograph monitor (SedLine sedation monitor; Masimo), respectively. The O3 regional oximetry recorded bilateral rSO_2_ by near-infrared spectroscopy, and the SedLine sedation monitor recorded the Patient State index. A 22-gauge catheter was placed into a radial artery and connected to a pressure transducer to monitor the arterial blood pressure, stroke volume variation and continuous cardiac index using the Vigileo system (Edwards Lifescience, Irvine, CA) with an arterial pressure waveform analysis sensor (FloTrac Sensor; Edwards Lifesciences).

#### 2.3.1. Anesthetic management.

Before the induction of anesthesia, an epidural catheter was placed in patients planning to undergo lobectomy or segmentectomy. General anesthesia was induced with remifentanil and propofol (1.0–2.0 mg/kg) in the desflurane group and with remifentanil and target-controlled infusion of 2.0 to 5.0 µg/mL plasma concentration of propofol, calculated with Diprifusor (Astra-Zeneca Pharmaceuticals, Macclesfield, UK) in the propofol group. After muscle blockade with rocuronium, a left-sided double lumen tube was intubated endotracheally and placed using fiberscope. The patients were placed in the lateral decubitus position while bending the surgical table to widen the intercostal space. Anesthesia was maintained with remifentanil and either propofol or desflurane. The doses of propofol and desflurane were gradually adjusted to a Patient State index of 25 to 50. Hemodynamic management was performed based on the Vigileo monitor, and the mean arterial blood pressure (MAP) was maintained within 20% of the preinduction value by the administration of phenylephrine, ephedrine, and fluids.

#### 2.3.2. Respiratory settings.

The subjects were mechanically ventilated with a mixture of air and oxygen at FiO_2_ 0.4. An anesthesia workstation was used (Dräger Perseus A500; Dräger, Lübeck, Germany), and the subject was ventilated with the AutoFlow-volume control ventilation mode. The tidal volume was set at 8 mL/kg (predicted body weight), and the positive end-expiratory pressure was set at 5 cmH_2_O. The respiration rate was adjusted to maintain the arterial partial pressure of carbon dioxide (PaCO_2_) at approximately 40 mmHg. After the pleural incision was made, OLV was initiated with an FiO_2_ of 0.4, and the tidal volume was not changed unless the peak airway pressure exceeded 30 cmH_2_O. The FiO_2_ was adjusted to maintain an SpO_2_ of 92% to 94%, and the respiration rate was adjusted to maintain PaCO_2_ at 40 mmHg. When the SpO_2_ dropped to <90%, the FiO_2_ was temporarily increased to 1.0 and then decreased after the cause of the drop was corrected. Unless a left-right difference in rSO_2_ or a sudden decrease in rSO_2_ was observed, FiO_2_ did not increase in response to a decrease in rSO_2_. Epidural bolus was not administered until the end of the study.

#### 2.3.3. Measurement values and time points.

**Figure 1.**
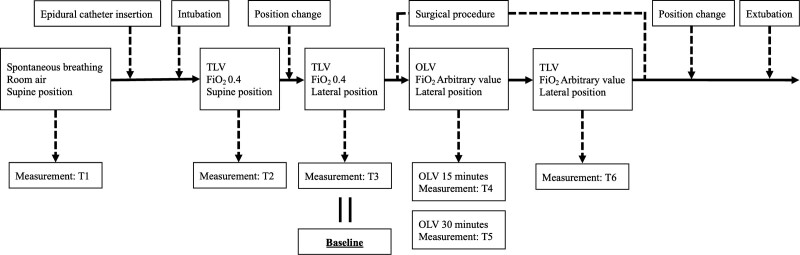
Measurement time points. T1: After arrival in the operating room. T2: Mechanical two-lung ventilation just before position change. T3 (baseline): Just before the start of surgery. T4: 15 minutes after the initiation of one-lung ventilation. T5: 30 minutes after the initiation of one-lung ventilation. T6: Two-lung ventilation at the end of surgery. FiO_2_ = fraction of inspiratory oxygen, OLV = one-lung ventilation, TLV = two-lung ventilation.

The measurement values included FiO_2_, heart rate, MAP, SpO_2_, body temperature, stroke volume variation and continuous cardiac index, Patient State index, left rSO_2_ (Lt. rSO_2_), right rSO_2_ (Rt. rSO_2_), concentration of propofol, end-tidal desflurane, concentration of remifentanil, and arterial blood gas sampling data (pH, arterial partial pressure of oxygen: PaO_2_, PaCO_2_, base excess, hematocrit). The measurements were performed at the following time points: after arrival in the operating room (T1); during mechanical two-lung ventilation just before position change (T2); just before the start of surgery (T3); 15 minutes after the initiation of OLV (T4); 30 minutes after the initiation of OLV (T5); during two-lung ventilation at the end of surgery (T6) (Fig. 1). We set the rSO_2_ value of T3 as the baseline value. Cerebral desaturation was defined as a relative decrease of ≥20% from the baseline rSO_2_ value or an absolute rSO_2_ value of <50%. The patient’s dominant hand was confirmed at the time of informed consent, and the dominant and nondominant sides were defined.

All complications during hospitalization were recorded.

### 2.4. Outcomes

The primary outcome was the absolute value of the decrease in the mean left and right rSO_2_ values from T3 to T5. The secondary outcomes were the absolute value of the decrease in the rSO_2_ value of the dependent and nondependent lung side from T3 to T5, the absolute value of the decrease in the rSO_2_ value on the dominant and nondominant side of the subject from T3 to T5, the number of cerebral desaturations and complications occurring until discharge from the hospital.

### 2.5. Statistical analyses

Prior to this study, a pilot study (decision number: 2018-210) was conducted to determine the sample size and collect results to select appropriate measurement values. In the pilot study, the absolute value of the decrease in the mean left and right rSO_2_ values from T3 to T5 was 14.0% in the propofol group and 9.8% in the desflurane group. Because the standard deviations (SDs) were 4.14 and 3.15, respectively, the SD was conservatively set at 4.14 in both groups. To detect a difference of 4.2% in the absolute value of the decrease in rSO_2_ between the propofol and desflurane groups by a *t* test with a detection power of 80% and at a 0.05 two-sided significance level, 16 cases per group (32 cases in total) were deemed necessary. We estimated that 10% of cases for per group would have unusable data, so we determined that 36 participants in whom measurements could be performed at least once during OLV were required. The full analysis set included all patients who received the protocol treatment at least once, except for those cases that were never evaluated for efficacy.

Baseline characteristics and aggregated data are presented as the median (interquartile range: IQR) or mean ± SD for continuous variables and numbers (%) for categorical variables. The patient hemodynamic parameters and results of arterial blood gas analyses at T3 and T5 were compared between the 2 groups using Student *t* test. The primary outcome was analyzed according to the intent-to-treat principle with an analysis of covariance (ANCOVA) adjusted for the mean left and right rSO_2_ value at T3 (baseline). The secondary outcomes were analyzed with adjustment for the baseline value using a similar model to that used for the primary outcome. All outcomes at each measurement point were described using the mean, median, SD and 25th and 75th percentiles, and the 95% confidence interval was calculated. The Fisher exact test was used to compare the incidence of cerebral desaturation. Imputation was not used for missing data for any outcomes, as no data were missing. Subgroup and post hoc analyses were not performed.

All statistical analyses were performed with the R software program, version 4.0.3 (www.r-project.org). Two-sided *P* value of <.05 were considered statistically significant. There was no adjustment for multiple comparisons, as the analyses of secondary endpoints were interpreted as exploratory.

## 3. Results

Between March and October, a total of 99 participants were screened for eligibility. Four patients scheduled for surgery at the same time on the same day were excluded. Seven patients were excluded because of a past medical history of postoperative nausea and vomiting, and 9 patients were excluded for other reasons (chronic kidney disease in 3, atrial fibrillation in 2, thoracic empyema in 2, sequelae after resection of a metastatic brain tumor in 1, and another clinical trial in progress in 1).

**Figure 2.**
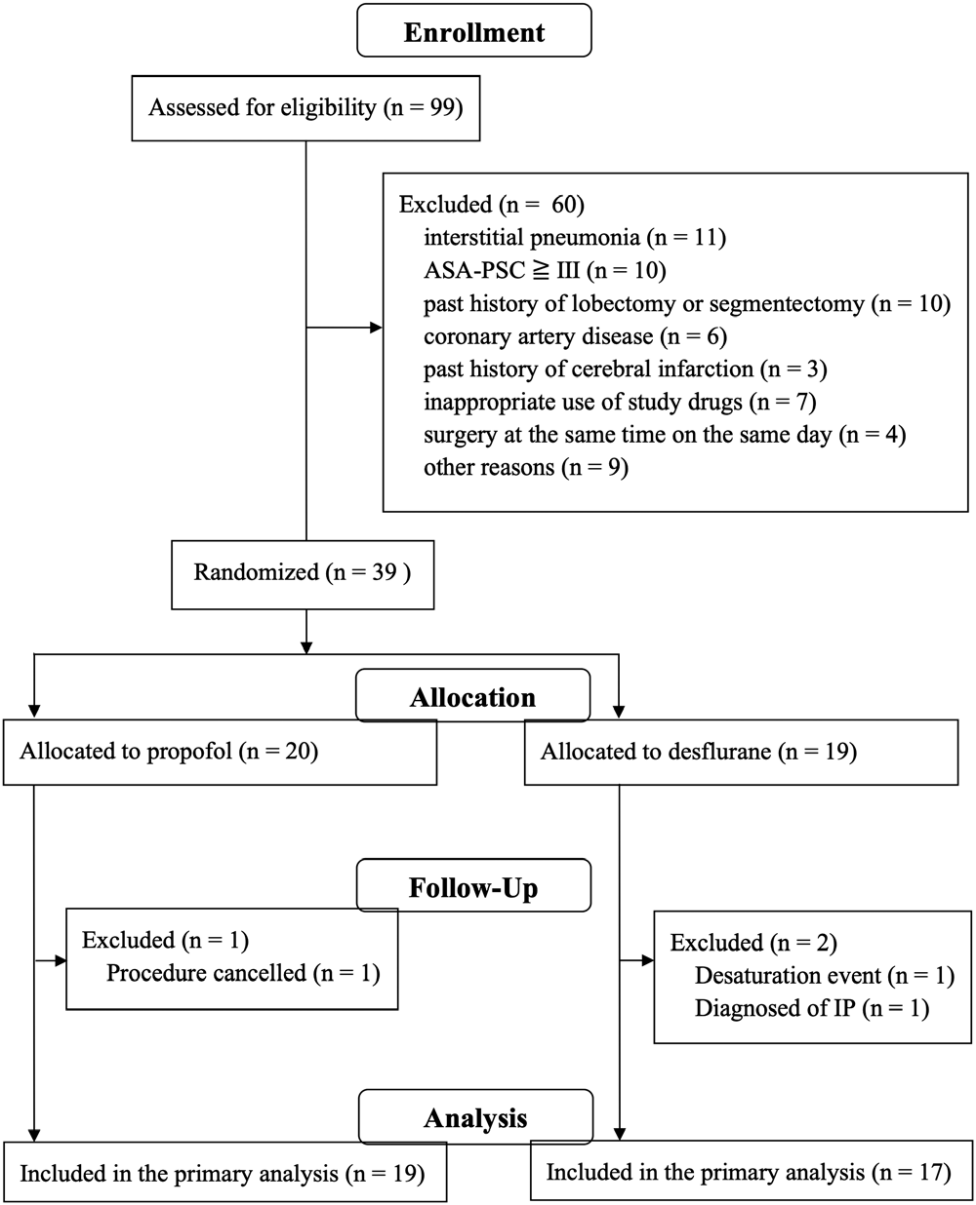
Figure 2. CONSORT diagram of the study flow. ASA-PSC = American Society of Anesthesiologists physical status classification.

Of the remaining 39 participants, 20 patients were assigned to the propofol group and 19 to the desflurane group (CONSORT Flow Diagram, Figure 2). One patient in the propofol group and 2 in the desflurane group were excluded after randomization (canceled surgery, n = 1; diagnosis of interstitial pneumonia after the induction of anesthesia, n = 1; and severe desaturation event occurring immediately after the induction of anesthesia, n = 1). Thus, a total of 36 patients (propofol group, n = 19; desflurane group, n = 17) thus completed the study protocol, and their datasets were available for the analysis.

**Figure 3.**
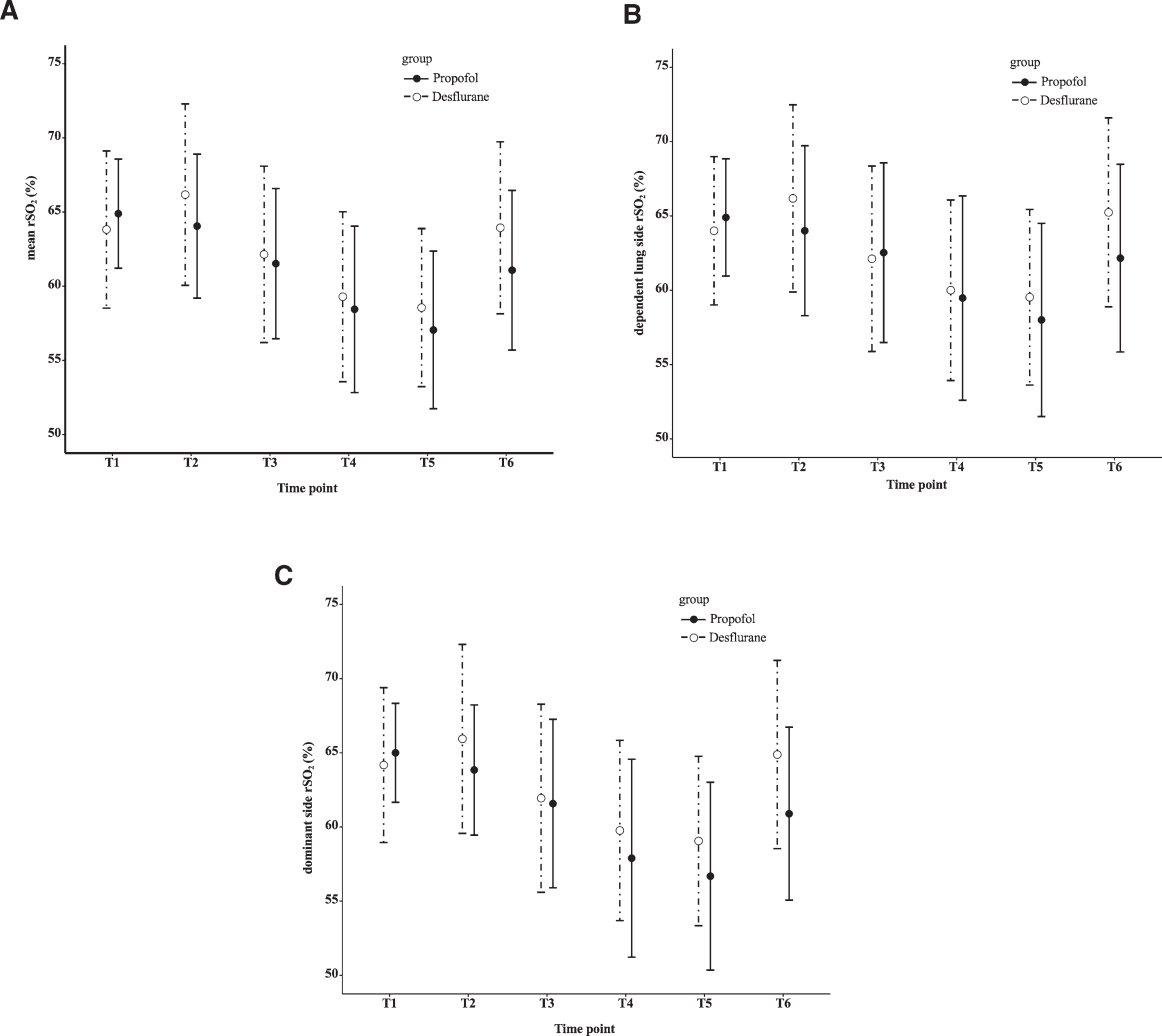
Changes in (A) mean rSO_2_, (B) dependent lung side rSO_2_, and (C) dominant side rSO_2._ Each value represents the mean ± standard deviation. The solid circle and solid line represent the propofol group, and the open circle and interrupted line represent the desflurane group. See Figure 1. for the definitions of the measurement time points. dependent lung side rSO_2_ = rSO_2_ values on the dependent lung side, dominant side rSO_2_ = rSO_2_ values on the dominant side, mean rSO_2_ = mean of the left and right rSO_2_, rSO_2_ = regional cerebral oxygen saturation.

The baseline characteristics and intraoperative variables of these 36 patients are shown in Table 1. Figure 3A shows the mean rSO_2_ values at each measurement point: 61.5% ± 5.1% in the propofol group and 62.2% ± 6.0% in the desflurane group at T3, and 57.1% ± 5.3% in the propofol group and 58.6% ± 5.3% in the desflurane group at T5. There was no significant difference between the groups in the absolute value of the decrease in rSO_2_ adjusted by the rSO_2_ value at T3 (Table 2).

**Table 1:** Characteristics of the patients.

Variable	Propofol group (n = 19)	Desflurane group (n = 17)
Male/Female	11 (57.9)/8 (42.1)	8 (47.1)/9 (52.9)
Age (y)	71 (67–78)	69.0 (63–73)
Body height (cm)	162 (152–167)	162 (154–166)
Body weight (kg)	60 (52–67)	54 (47–59)
ASA-PSC (Ⅰ/Ⅱ)	0/19	3 (17.6)/14 (82.4)
FVC (% predicted)	109 (95–119)	117 (111–125)
FEV_1.0_/FVC (%)	74 (67–79)	75 (63–79)
%FEV_1.0_ (%)	104 (93–118)	104 (99–115)
Preoperative SpO_2_ (%)	97 (96–98)	97 (96–97)
Hugh-Jones grade (Ⅰ/Ⅱ)	13 (68.4)/6 (31.6)	11 (64.7)/6 (35.3)
Smoking history	12 (63.2)	11 (64.7)
Lobectomy/segmentectomy/wedge resection/mediastinal tumor/pleural tumor	6 (31.6)/3 (15.8)/9 (47.4)/1 (5.3)/0	6 (46.2)/4 (23.5)/5 (29.4)/1 (5.9)/1 (5.9)
Operation side (left/right)	9 (47.4)/10 (53.6)	9 (52.9)/8 (47.1)
Infused fluid (mL)	1550 (1100–1825)	1200 (1050–1650)
Urinary output (mL)	250 (165–690)	400 (160–670)
Estimated blood loss (mL)	10 (5–15)	10 (5–30)
Duration of anesthesia (min)	246 (198–324)	243 (192–322)
Duration of OLV (min)	138 (90–220)	134 (76–224)
Duration of surgery (min)	152 (110–243)	163 (105–244)
Dominant side (left/right)	0/19	0/17

Statistics presented: median (interquartile range) for continuous variables; n (%) for categorical variables.

%FEV_1.0_ = percent predicted forced expiratory volume in 1 second, ASA-PSC = American Society of Anesthesiologists physical status classification, FVC = forced vital capacity, FEV_1.0_ = forced expiratory volume in one second, OLV = one-lung ventilation, SpO_2_ = percutaneous oxygen saturation.

**Table 2:** Primary and secondary outcomes: ANCOVA adjusted for the value of rSO_2_ at the T3.

Outcome	Variable	β*	95% CI	*P* value
Mean of left and right rSO_2_ (primary endpoint)	Group	0.95	−0.32, 2.20	.15
	rSO_2_ at T3	0.10	−0.02, 0.21	.12
rSO_2_ on the dependent lung side	Group	1.90	0.22, 3.60	.03
	rSO_2_ at T3	0.07	−0.07, 0.21	.32
rSO_2_ on the nondependent lung side	Group	−0.07	−1.40, 1.30	.92
	rSO_2_ at T3	0.06	−0.06, 0.17	.32
rSO_2_ on the dominant side	Group	2.00	0.39, 3.70	.02
	rSO_2_ at T3	0.08	−0.06, 0.22	.27
rSO_2_ on the nondominant side	Group	−0.20	−1.60, 1.20	.79
	rSO_2_ at T3	0.05	−0.07, 0.17	.43

*β is the coefficient obtained from ANCOVA. If β of group is >0, this indicate that the absolute value of the decrease in rSO_2_ from T3 to T5 is greater in the Propofol group than Desflurane group, with adjustment for rSO_2_ at T3.

CI = confidence interval, rSO_2_ = regional cerebral oxygen saturation, T3 = just before the start of surgery, T5 = 30 minutes after initiation of one-lung ventilation.

After adjustment by the rSO_2_ value at T3, the absolute value of the decrease in rSO_2_ was significantly greater in the propofol group than in the desflurane group on both the dependent lung and the dominant sides (Table 2). Figure 3B and C shows the rSO_2_ values on the dependent lung and dominant lung sides, respectively, in both groups. The PaO_2_ value at T3 was significantly higher in the desflurane group. There were no significant differences in the MAP, continuous cardiac index, SpO_2_, PaCO_2_, any other hemodynamic parameters or the arterial blood gas data between the groups at T3 and T5 (Table 3). Furthermore, there were no significant differences in the incidence of cerebral desaturation events between the groups (propofol group, n = 5; desflurane group, n = 2; *P* = .41). One patient in each group showed a decrease in SpO_2_ immediately after the start of one-lung ventilation due to malposition of the double lumen tube, and the FiO_2_ was temporarily increased to 1.0 to adjust the tube position using a fiberscope. No cases required the adjustment of FiO_2_ in response to decreased rSO_2_. Complications occurred in 2 subjects in each group: paroxysmal atrial fibrillation and postoperative respiratory failure requiring admission to the intensive care unit in one patient each in the propofol group and prolonged pulmonary leakage and continuous atrial fibrillation requiring electrical ablation in the desflurane group.

**Table 3:** Intraoperative anesthetic variables and arterial blood gas data.

		Propofol group (n = 19)	Desflurane group (n = 17)	*P* value
T3	FiO_2_	0.40 (0)	0.40 (0)	NA
	HR (beat/min)	58.7 (5.3)	58.7 (7.2)	.99
	MAP (mmHg)	90.4 (11.4)	85.6 (21.6)	.42
	SpO_2_ (%)	99.4 (0.6)	99.3 (0.7)	.58
	BT (℃)	36.6 (0.4)	36.7 (0.4)	.85
	pH	7.40 (0.03)	7.42 (0.02)	.13
	PaO_2_ (mmHg)	168.8 (30.5)	190.7 (25.8)	.03
	PaCO_2_ (mmHg)	40.2 (1.7)	40.8 (1.7)	.87
	Hematocrit (%)	37.9 (5.2)	37.1 (3.7)	.62
	Cardiac index (L/min/m^2^)	2.5 (0.5)	2.5 (0.3)	.92
	Patient State index	33.8 (6,3)	31.7 (5.6)	.29
	Ce. Remifentanil (ng/mL)	3.2 (0.5)	3.2 (0.7)	.88
	Ce. Propofol (mcg/mL)	2.5 (0.5)		
T5	FiO_2_	0.33 (0.21)	0.31 (0.12)	.72
	HR (beat/min)	68.4 (9.3)	65.0 (9.4)	.72
	MAP (mmHg)	89.1 (9.2)	89.2 (6.3)	.29
	SpO_2_ (%)	93.3 (1.1)	93.3 (0.7)	.96
	BT (℃)	36.4 (0.4)	36.4 (0.3)	.63
	pH	7.41 (0.02)	7.42 (0.02)	.50
	PaO_2_ (mmHg)	58.6 (12.3)	62.9 (3.3)	.16
	PaCO_2_ (mmHg)	40.2 (1.7)	40.7 (1.7)	.85
	Hematocrit (%)	38.2 (5.4)	37.9 (4.1)	.87
	Cardiac index (L/min/m^2^)	2.7 (0.4)	2.7 (0.4)	.79
	Patient State index	33.1 (6.1)	31.3 (5.4)	.36
	Ce. Remifentanil (ng/mL)	3.5 (0.9)	3.2 (0.7)	.26
	End-tidal desflurane (%)		3.9 (0.2)	

Satistics presented: mean (SD).

There were not significant different in any values between the 2 groups at T3 and T5, respectively.

BT = body temperature, Ce = effect site concentration, FiO_2_ = fraction of inspiratory oxygen, HR = heart rate, MAP = mean arterial blood pressure, PaCO_2_ = arterial partial pressure of carbon dioxide, PaO_2_ = arterial partial pressure of oxygen, SpO_2_ = percutaneous oxygen saturation, T3 = just before the start of surgery, T5 = 30 minutes after initiation of OLV.

All patients were discharged with no central neurological complications.

## 4. Discussion

The present study was performed to compare the effects of propofol and desflurane on cerebral oxygenation evaluated by rSO_2_ during OLV while avoiding a high FiO_2_ level. There were no significant differences between the 2 groups in the absolute value of the decrease in the mean rSO_2_ up to 30 minutes with an SpO_2_ of 92% to 94% after the initiation of OLV. We hypothesized that cerebral oxygenation would be more markedly impaired in the propofol group than in the desflurane group because of the greater reduction in the CBF/CMRO_2_ ratio by propofol. However, the present study indicated that propofol anesthesia was comparable to desflurane anesthesia with regard to cerebral oxygenation during OLV. Thus, both anesthetic agents can be safely used in routine clinical practice in patients who do not have symptomatic cerebrovascular or cardiovascular complications.

Desaturation events during OLV can easily occur due to tube misalignment and tube obstruction with sputum. Thus, mechanical ventilation tends to be maintained at a higher FiO_2_ level to avoid desaturation events during OLV. However, a high FiO_2_ causes oxidative stress and is dose-dependently associated with respiratory complications and 30-day mortality.^[2,3]^ Furthermore, hyperoxemia can carry a risk of myocardial ischemia, reduced cardiac output, reduced coronary blood flow, and reduced renal blood flow.^[20]^ The optimal ranges of target FiO_2_ and SpO_2_ during general anesthesia for major surgery are unclear.^[21]^ To prevent lung injury during OLV due to a high FiO_2_, the FiO_2_ should be kept as low as possible to maintain an SpO_2_ of ≥ 90%. In the present study, the SpO_2_ was maintained at 92% to 94% during OLV without the need for a high FiO_2_ (FiO_2_ values at T5: 0.33 ± 0.21 in the propofol group and 0.31 ± 0.12 in the desflurane group; SpO_2_ values at T5: approximately 93% in both groups). In general, the MAP, PaO_2_, and PaCO_2_ are closely related to cerebral perfusion and oxygenation. In the present study, the PaO_2_ values in the desflurane group were significantly higher than those in the propofol group at T3. However, the MAP and PaCO_2_ values did not differ to a statistically significant extent, and PaCO_2_ was maintained at approximately 40 mmHg in both groups at T3 and T5. In general, CBF is maintained when PaO_2_ is ≥60 mmHg. Therefore, the difference at T3 is not expected to affect CBF. This suggests that maintaining normocapnia and other hemodynamic parameters without significant fluctuations may prevent a significant reduction in rSO_2_, even under propofol anesthesia.

Although both volatile anesthetics and propofol reduce the CMRO_2_ value, the effects on CBF differ between the 2 agents. Propofol reduces the CBF more markedly than the CMRO_2_, resulting in an overall reduction in the CBF/CMRO_2_.^[12–15]^ In contrast, volatile anesthetics reduce the CMRO_2_ in a dose-dependent manner, and the CBF increases, depending on the MAC after maximal cerebral metabolic suppression, which mainly occurs after exceeding 1 MAC.^[16–18,22]^ However, the effect on the CBF is unchanged or even slightly reduced with a low MAC.^[22]^ We previously demonstrated that 0.5 MAC of desflurane had no significant vasodilatory effect on the diameter of rats’ cerebral pial arterioles.^[23]^ Furthermore, Mielck et al^[24]^ reported that 1 MAC desflurane significantly reduced the CBF in comparison to an awake state in healthy humans. In the present study, the end-expiratory desflurane concentration was 3.9 ± 0.2%, which was <1 MAC, even considering the age of the participants. Therefore, cerebrovascular dilation might not have occurred as expected, and no marked difference in the rSO_2_ reduction was noted between the desflurane and propofol groups.

Important factors associated with the cerebral oxygen supply are arterial oxygen saturation (SaO_2_), hemoglobin, cardiac output, and CBF. The cerebral oxygen demand during general anesthesia depends on the body temperature, seizure, type of anesthetic agent, and depth of anesthesia. Although oxygen dissolves directly in plasma depending on the PaO_2_ value, the amount is very small, and its role in the oxygen supply is minimal. The rSO_2_ is calculated by measuring the hemoglobin in arterial, venous, and capillary blood, assuming that 70% of the venous blood and 30% of the arterial blood are included in the cerebral blood volume.^[25,26]^ Masimo O3 also calculates the rSO_2_ under this assumption, providing a close correlation with the reference cerebral oxygen saturation (0.3 SaO_2_ + 0.7 SjO_2_), as measured by blood gas sampling.^[26]^ Although the rSO_2_ reflects the cerebral tissue oxygenation and blood flow, the rSO_2_ is more closely associated with SjO_2_ than SaO_2_ due to the high proportion of venous components.^[25]^ Our study showed no marked changes in hemodynamic parameters from T3 to T5, suggesting that a decrease in rSO_2_ reflected a decrease in SaO_2_.

The present study showed that the absolute value of the decrease in rSO_2_ was significantly larger in the propofol group than in the desflurane group on both the dependent lung and dominant sides. The bilateral SjO_2_ values did not differ to a statistically significant extent in healthy patients in the supine position; however, postural change during anesthesia, such as adopting the Trendelenburg or beach chair positions, generally affects cerebral circulation.^[27,28]^ In the present study, patients were placed in the lateral decubitus position with bending of the surgical table. The cerebral blood volume is presumed to increase on the dependent lung side while in the lateral decubitus position.^[27]^ Blauenstein et al^[29]^ reported that in healthy right-hand dominant patients, the CBF in the inferior frontal region is higher in the right hemisphere than in the left hemisphere. Although it has been reported that midazolam decreases the CBF in the left prefrontal cortex, we found no evidence that propofol or desflurane affects the laterality of the cerebral blood volume.^[30]^ The significant difference in the rSO_2_ reduction between the 2 groups on both the dependent lung and dominant sides may be induced an increase in the cerebral blood volume or CBF because propofol has no vasodilatory effect. However, evidence regarding the laterality of cerebral oxygenation measured by the near-infrared spectroscopy system is insufficient to explain our results at present, and the degree of difference between the 2 groups is not an issue in clinical practice.

The present study was associated with several limitations. First, our study did not measure the CBF or CMRO_2_, so we were unable to determine whether the change in rSO_2_ was due to a change in the blood flow or a decrease in SaO_2_. While the intracranial pressure may also influence the CBF, this impact cannot be ruled out, as it was not measured in this study. Second, we did not strictly protocolize the phenylephrine and ephedrine doses, although the MAP was controlled. Because phenylephrine reduced the rSO_2_ value due to extracranial blood flow, our results might not solely reflect frontal lobe oxygenation.^[31]^ Third, since postoperative cognitive dysfunction was not evaluated, the effect of a decreased rSO_2_ on the neurological prognosis was unclear.

In conclusion, our present study suggests that both propofol and desflurane anesthesia can maintain comparable cerebral oxygenation during OLV under an SpO_2_ of 92% to 94% and normocapnia settings in patients with no serious complications. As only patients with a healthy brain function were included in this study, cerebral oxygenation monitoring may be necessary in patients at risk for cerebrovascular disorders, such as cerebrovascular disease and cardiovascular disease.

## Author contributions

Conceptualization: Keishu Hayashi, Kumiko Tanabe, Hiroki Iida.

Data curation: Keishu Hayashi.

Formal analysis: Takuma Ishihara.

Funding acquisition: Hiroki Iida.

Investigation: Keishu Hayashi, Yuko Yamada.

Methodology: Kumiko Tanabe, Hiroki Iida.

Project administration: Keishu Hayashi, Hiroki Iida.

Supervision:Hiroki Iida.

Writing-original draft: Keishu Hayashi, Yuko Yamada, Takuma Ishihara, Kumiko Tanabe.

Writing-review and editing: Kumiko Tanabe, Hiroki Iida.

All authors read and approved the final articles.
